# Interrupted processing of paired somatosensory stimuli in short interstimulus intervals: role of thalamo-cortical oscillations

**DOI:** 10.55730/1300-0144.5444

**Published:** 2022-03-13

**Authors:** Nermin TEPE, Hürrem Evren BORAN, Salih GEDÜK, Ergin DİLEKÖZ, İlkay ULUSOY, Hayrunnisa BOLAY

**Affiliations:** 1 Department of Neurology, Balıkesir University Faculty of Medicine, Balıkesir, Turkey; 2 Neuropsychiatry Centre, Gazi University Faculty of Medicine, Ankara, Turkey; 3 Department of Neurology, Gazi University Faculty of Medicine, Ankara, Turkey; 4 Department of Electrical and Electronics Engıneerıng, Middle East Technical University, Ankara, Turkey; 5 Department of Pharmacology, Gazi University Faculty of Medicine, Ankara, Turkey

**Keywords:** Paired-pulse somatosensory evoked potentials, interstimulus interval, thalamus, high-frequency oscillations, sensory gating

## Abstract

**Background/aim:**

Discrimination of consecutive sensory stimuli is imperative for proper sensory perception and behavioral response. We aimed to investigate the emergence of paired somatosensory responses in relation to the interstimulus interval (ISI) change.

**Materials and methods:**

Paired stimulus with 35 ms, 50 ms, 80 ms, 140 ms, and 500 ms ISI was applied to the median nerve and evoked responses were recorded from the primary somatosensory cortex in rats. Early and late components of both responses were analyzed in different frequency bands.

**Results:**

The amplitudes were comparable for the 1st responses (S1), while the amplitude of the 2nd responses (S2), and S2/S1 sensory gating ratio were significantly lower at 35 and 50 ms ISI values. S2/S1 ratio was close to 1 at 500 ms ISI. The duration and latency of the 2nd response was also different at 35 ms ISI. In the 2nd responses, area of early high-frequency oscillations (150–400 Hz) was significantly lower at 35 ms ISI values.

**Conclusion:**

The shaping of 2nd somatosensory response is dependent on ISIs. Early high-frequency oscillations changes without accompanying late high-frequency oscillations alterations, may indicate that reduced thalamo-cortical drive to the cortex take a part in determining the 2nd response at short ISI. Further research is required by using neuropsychiatric disorder models where somatosensory perception is impaired.

## 1. Introduction

In sensory processing, recognition of consecutive stimuli and formation of appropriate responses are related to the time between the first stimulus. In case of dual or multiple stimuli, the magnitude of the cortical potentials arising due to peripheral stimulus is thought to be dependent on interstimulus interval (ISI). However, the electrophysiological mechanisms of resulting habituation from tactile stimuli remain elusive; examining changes in the somatosensory cortex by changing ISI is a valid method to investigate somatosensory habituation [1]. In recent years, paired-pulse stimulation technique has been widely utilized to demonstrate intracortical inhibition or facilitation of the cortical excitability and plasticity [2].

In paired-pulse peripheral nerve stimulation, the first stimulus activates the excitatory pathways, while the second stimulus is suppressed due to the activation of the inhibitory interneuronal pathways. It has been shown that the inhibition between neurons of the primary sensory cortex, thalamus, prefrontal cortex, hippocampus, and the rhinal cortex plays a role in sensory gating [3]. Recently, Boran et al. reported a significant prolongation of somatosensory temporal discrimination during attacks in patients with episodic migraine [4]. Furthermore, Vuralli et al. showed a marked prolongation of the somatosensory temporal discrimination in chronic migraine [5].

Each moment, a large flow of data input occurs in the brain. Some of these data may be related to the current status, while others may not. The selection of the important input in the current status is called “sensory gating”. The gating ratio reflects the extent of attenuation in the second signal [6]. Since sensory gating has not been examined at very different interstimulus intervals at the same time, we wanted to learn about its physiology by looking at different intervals such as 35, 50, 80,140, and 500 ms. For this reason, we wanted to investigate the basal physiological changes in these intervals in terms of amplitude, latency, duration, area and contribution of HFOs. So we designed this study by recording somatosensory evoked potential (SSEP) changes from the primary somatosensory cortex by applied two stimulation of the median nerve at different ISIs in rats under general anesthesia. The purpose was to analyze obtained SSEP data to observe the change in sensory gating in different ISIs and to obtain more detailed insights into sensory gating.

## 2. Materials and methods

### 2.1. Animals and surgical procedure

After approval from the Local Ethics Committee for Animal Experimentation Gazi University (no. 18/042), twelve Wistar rats weighing between 200 and 250 g were included in the study. Urethane (1200 mg/kg, i.p.) was used for general anesthesia and the depth of general anesthesia was adjusted based on, respiration, and hind-paw pinch reaction. Animals were placed on heating blankets with continuous monitoring of the rectal temperature after the desired depth of anesthesia was achieved. The head was placed in a stereotactic frame for stabilization, after which a median incision was made on the head skin and a circular hole of approximately 2 mm in diameter was opened in the right parietal area with preserving the dura mater by using a drill.

### 2.2. SSEP recordings and data analysis

Bipolar glass electrodes were placed on the area corresponding to the somatosensorial cortex (2.6 mm rostral, 5.5 mm lateral to bregma) [7]. Also, Ag/AgCl ground electrode was placed subcutaneously in the neck and subdermal needle electrodes for electrical stimulation were placed in the distal forelimbs. EEG amplifier (Kaldiray, EX-2C, YSED, Ankara, Turkey) was used for signal amplification. EEG signals were filtered between 0.05 and 100 Hz and sampled at 1 kHz. Evoked potentials in the primary somatosensory cortex were recorded and analyzed by using a data collection system (Powerlab 8/s; AD Instruments, New South Wales, Australia). The paired-pulse stimulation was applied to the distal forelimbs with a square-wave electrical pulse having a duration of 0.2 milliseconds (ms). The paired-pulse stimulation rate was 0.1 Hz. Recordings were obtained at interstimulus intervals of 35, 50, 80, 140, and 500 ms. Twenty recordings were averaged for each ISI. The peak-to-peak amplitudes, latencies, durations, and areas of 1st responses (S1) and 2nd responses (S2) from data collected in each recording were measured for each ISI. Additionally, the ratio between the amplitudes of S2 and S1 responses was determined as S2/S1 gating ratio for each ISI. The data were also analyzed at different frequency bands (7–13 Hz, 14–50 Hz, 51–150 Hz, 150–400 Hz, and 400–800 Hz), including the high-frequency oscillations (HFO). The HFO analyses were performed using the MATLAB platform (Mathworks Inc.).

In the area analysis, the first step the raw data was averaged over twenty records. The signal is analyzed by considering 4 regions. The first region is between the onset and the peak latency first response, the second region starts with the peak latency first response and the end of the first response. The third region is between the onset and the peak latency second response, forth region starts with the peak latency of second maximum and the end of the second response. In human SEP studies, the area between the beginning of the first response and the peak latency was taken for early HFO, while the area between the peak latency of the first response and the end of the response was taken for late HFO.

**Figure 1:**
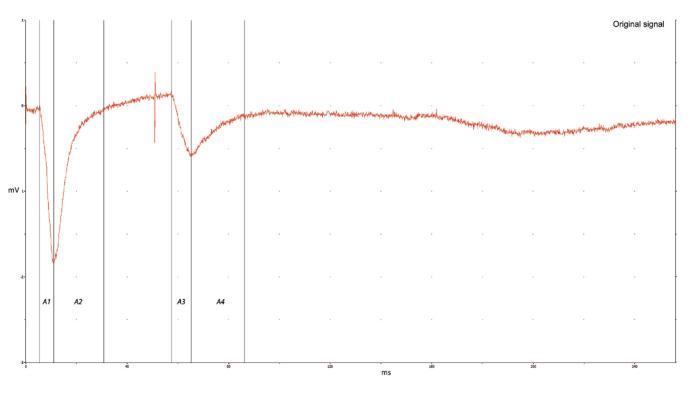
Area calculation in at 50 ms ISI sample; thalamo-cortical response A1 and cortical response A2 in the primary response, and thalamo-cortical response A3 and cortical response A4 in the second response according to onset and negative peaks.

Separation of early and late component of HFOs by the borderline as N20 peak or N20 m peak was proposed by Nakano and Hashimoto [8] (Figure1). The first and third regions are corresponding thalamo-cortical response while the second and fourth regions are cortical. To evade the undesired effects (stimulation artefacts) due to stimulation, the first 3 ms after the stimulation time was not used in the signal processing. The stimuli time for signal processing was taken as the 3 ms after the exact stimuli time. Later, the data was band-pass filtered to get the information for various frequency intervals. The filter was ideal filter which is a built-in method in MATLAB. Then, the area of the corresponding regions was calculated, for each output signal of the ideal filters.

### 2.3.Statistical analysis

Statistical Package for Social Sciences (SPSS) for Windows Version 21.0 software was used for statistical analyses. Transformation was made to normalize the distribution of the data. Results were expressed as mean ± standard deviation. According to ISI, the latencies, amplitudes, durations, and areas of the first and second responses and S2/S1 ratio were examined using one-way ANOVA. For the posthoc analyses regarding the change within ISIs, a Bonferroni test was utilized. Correlations were investigated using Spearman’s test.

In detailed area analysis, in order to get a better statistical analysis between the calculated energy values, the undesired effect of measurements should have been omitted. Therefore, the area of each region and frequency band was divided by the total area of the four regions. For statistical comparison, parametric two-sample t-test was conducted. p values <0.05 were considered statistically significant.

## 3. Results

**Figure 2:**
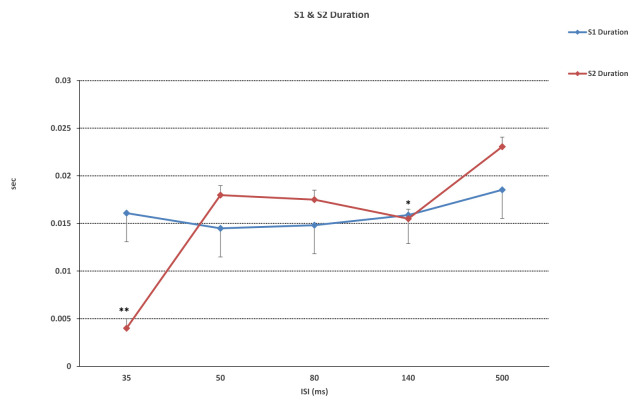
The duration of the first response (S1) was not significantly different from ISI, while the duration of the second response (S2) at 35 ms was significantly lower than 50 ms, 80 ms, 140 ms, 500 ms (**p < 0.001) and the duration of the S2 at 140 ms was significantly lower than 500 ms (*p = 0.004). ISI: interstimulus interval. One-way ANOVA test used, for the posthoc analyses regarding the change within ISIs, a Bonferroni test used.

**Figure 3:**
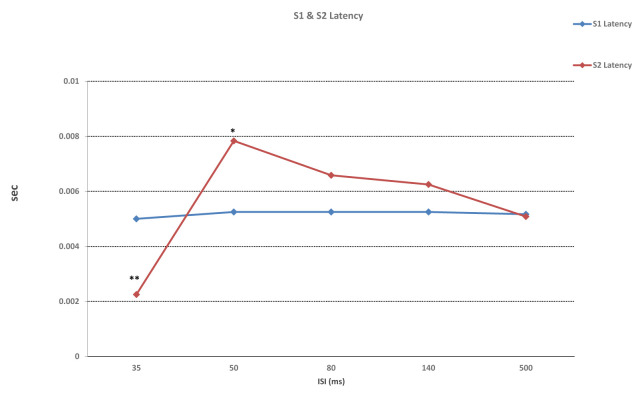
While there was no significant difference in latency with ISI in the first response (S1), a significant difference was found between 35 ms and 50 ms, 80 ms, 140 ms (**p < 0.001,), and 500 ms (p = 0.01) in the second response (S2). In addition, the latency of S2 at 50 ms was significantly different from 500 ms (*p = 0.02,). ISI: interstimulus interval. One-way ANOVA test used, for the posthoc analyses regarding the change within ISIs, a Bonferroni test used.

**Figure 4:**
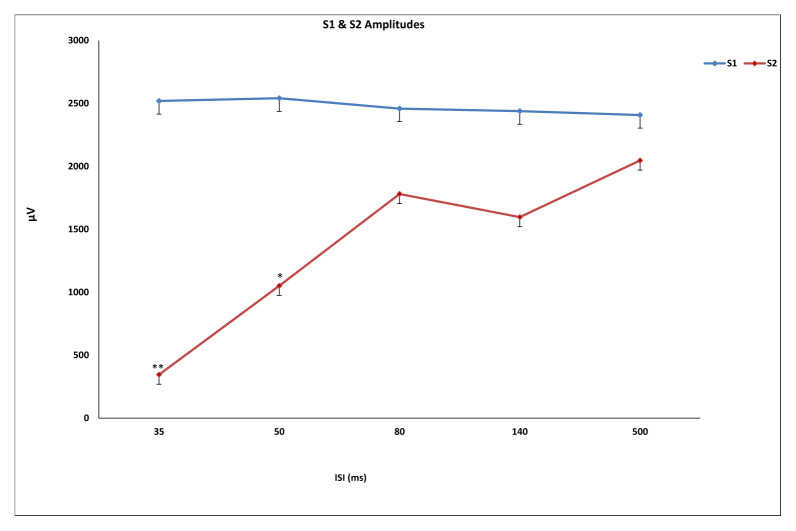
The amplitudes of the second response (S2) at 35 ms and 50 ms ISI were significantly lower than others (**p < 0.001,* p < 0.05,). No significant difference was found in the first response (S1) amplitudes with ISI values. ISI: interstimulus interval. One-way ANOVA test used, for the posthoc analyses regarding the change within ISIs, a Bonferroni test used.

**Figure 5:**
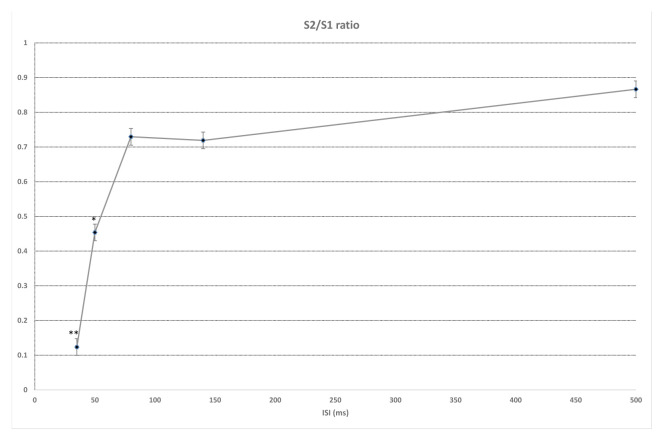
The amplitudes of S2/S1 ratio at 35 ms and 50 ms ISI were significantly lower than other ISI values (**p < 0.001, *p = 0.001,). The S2/S1 ratio approached 1 at ISI of 500 ms. ISI: interstimulus interval. One-way ANOVA test used, for the posthoc analyses regarding the change within ISIs, a Bonferroni test used.

**Figure 6:**
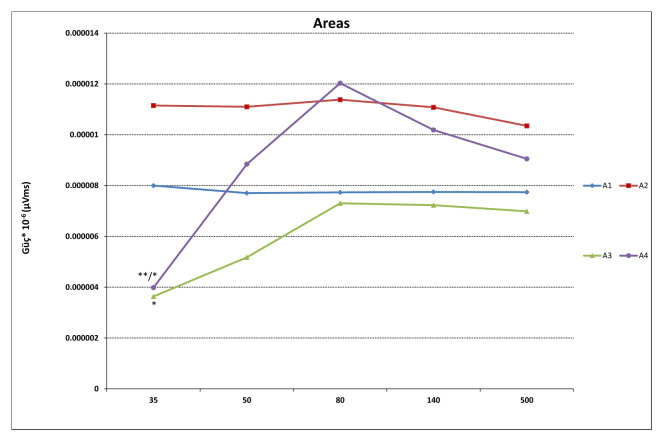
The graph showing the areas under the early and late components of the first (S1) and second (S2) responses. A1 and A3 denote predominant thalamo-cortical contribution, while A2 and A4 represent major cortical contribution. There was no difference in A1 and A2 areas of the first response with ISI. At ISI of 35 ms, A3 response was significantly lower than 140 ms (*p < 0.05) and A4 was significantly different from 50 ms, 80 ms, 140 ms (**p < 0.001), 500 ms (*p < 0.05). ISI: interstimulus interval. Parametric two-sample t-test used.

**Figure 7:**
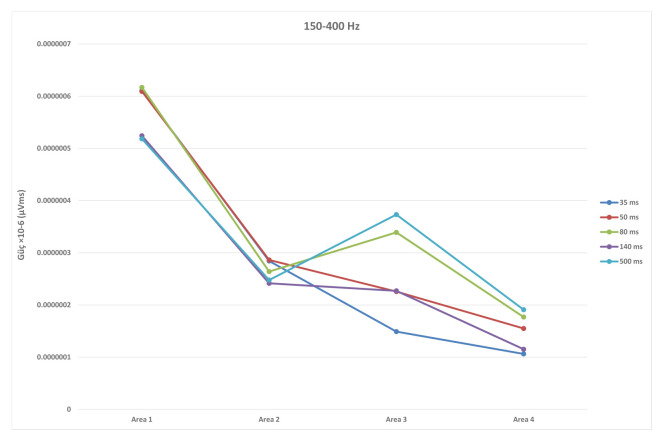
Evaluation of early (A1, A3) and late (A2, A4) components of paired responses in 150–400 Hz. There was a significant difference between 35 ms and 80 ms (p = 0.011), 35 ms and 500 ms (p = 0.002) in A3 at 150–400 Hz. ISI: interstimulus interval, HFO: high-frequency oscillations. Parametric two-sample t-test used.

While ISI for the duration of the first responses (S1) were not significantly different, significant differences in second responses (S2) were found (F (4, 55) = 21,887, p < 0.001). Significant differences between 35 ms and 50, 80, 140, and 500 ms were found in duration of S2 (p < 0.001), as well as between 140 ms and 500 ms (p = 0.004) (Figure 2). Similarly, while ISI did not differ significantly in S1 latency values, significant differences in S2 latency values were found (F (4, 55) = 12,492, p < 0.01). With regard to S2 latency values, significant differences between 35 ms and 50, 80, 140 ms (p < 0.001) and 500 ms (p = 0.015) were found, in addition to a significant difference between 50 ms and 500 ms (p = 0.02) (Figure 3). S1 amplitude did not differ significantly together with ISI, while S2 was found to change significantly with ISI (F (4, 55) = 23,554, p < 0.001). S2 amplitude with an ISI of 35 ms was significantly lower as compared to ISIs of 50, 80, 140, and 500 ms (p < 0.001). Also, significant differences between ISI of 50 ms and ISIs of 80 and 500 ms were found (p = 0.026, p = 0.003, respectively). ISIs of 80, 140, and 500 ms did not differ significantly (p > 0.005) (Figure 4). While the mean amplitude of the second response was found to decrease with lower ISIs, the mean amplitude of the second response was found to approach the first response at higher ISIs and the second to first response amplitude ratio was close to 1, particularly at an ISI of 500 ms. Also, S2/S1 sensory gating ratio differed significantly from ISI (F (4, 55) = 38,425, p < 0.001). An analysis of ISI subgroups showed significant differences between 35 ms and 50, 80, 140, and 500 ms (p < 0.001), as well as between 50 ms and 80, 140, and 500 ms (p = 0.001, p = 0.002, p < 0.001, respectively). On the other hand, no significant differences were found between ISIs of 80, 140, and 500 ms (p > 0.05) (Figure 5). When areas were assessed, no significant differences in the thalamo-cortical (A1) and cortical (A2) components of the first response were observed, as opposed to significant differences with ISIs in the thalamo-cortical component (A3) (F (4, 55) = 4569, p < 0.01) and cortical component (A4) of the second response (F (4, 55) = 8797, p < 0.01). Significant differences at ISIs of 35 ms and 140 ms were found in the A3 component (p = 0.003) and also, significant differences were observed between ISI of 35 ms and 50, 80, 140 (p < 0.001), and 500 ms (p = 0.04) in the A4 component (Figure 6). In addition, when the t-test is performed between A1 and A3 and A2 and A4, there is a significant difference between A1 and A3 (p = 0.004) and A2 and A4 (p < 0.001) at 35 ms, and between A1 and A3 (p < 0.001) at 50 ms. There was no significant difference between A2 and A4 (p > 0.005) at 50 ms. In the evaluation of different ISIs for areas of different frequency bands, it was found to be significant between 35 ms and 80 ms (p = 0.011), 35 ms and 500 ms (p = 0.002) in Area 3 at 150–400 Hz, while at 400–800 Hz no significance was found (Figure 7). In HFO analysis according to ISI, the second response (A43)/first response (A21) was statistically significant in 35, 50, 80, and 140 ms between 50–150 Hz and 150–400 Hz. On the other hand, statistical significance was not observed in any HFO at 500 ms displayed Table. Correlation was found between ISI and S2 amplitude (p < 0.001, r = 0.727), S2/S1 ratio (p < 0.001, r = 0.796), S2 duration (p < 0.001, r = 0.605), A3 area (p < 0.01, r = 0.439), and A4 area (p = 0.04, r = 0.367). S2/S1 ratio also correlated with the S2 amplitude (p < 0.01, r = 0.816).

## 4. Discussion

The processing of sensory information occurs at the primary somatosensory cortex, thalamus, and posterior parietal cortex. Thalamic burst firing plays a significant role in sensory gating and is activated in painful stimuli, with their inhibition causing alterations in the nociceptive responses [9]. High-frequency oscillations are believed as a generator in the formation of thalamo-cortical afferent fibers, and then in the formation of inhibitory interneurons in the primary cortical cortex (area 3b) [10]. Previously, shorter intervals in paired stimuli were found to be associated with decreased magnitude of the second stimulus (particularly at 25–75 ms). These responses were particularly more commonly observed in passive or paralyzed animals, while it was limited to 25 ms in whisking or active animals [11, 12]. In our study, the second sensory responses were found to be dependent on ISI values. Paired stimuli at 35, 50, 80, 140, and 500 ms intervals were causing no alterations in S1 while increasing ISI led to increasing S2 amplitudes. These exhibited more rapid and more significant increases up to ISI of 80 ms, after which an attenuation was observed. S2/S1 ratio was observed to approach 1 as ISI approached 500 ms. Parallel to this, the firing due to the second stimulus was suppressed following the first stimulus. The postexcitatory suppression has been demonstrated in the somatosensory cortex of cats and monkeys, and in the barrel cortex in rats [13,14]. While the inhibition mediated by the reticular cells of the thalamus after the first stimulus leads to hyperpolarization of the neurons in the ventral posteromedial nucleus causing the burst pattern, after 100 to 150 ms reduction of the inhibition during the second stimulus causes a burst response in the projection neurons of the thalamus and leading to depolarization [15]. Our observations that the second amplitude is approaching the first response with increasing ISI are supportive of these previous findings.

Previous systemic studies showed significant suppression of the second response at ISIs of 20–40 ms after paired-pulse stimulation of the median nerve in healthy individuals, no such suppression could be observed at ISI > 100 ms [16,17]. The marked suppression of the second response was suggested as short-term plasticity [18]. Similarly, in our study, a significant rise in the amplitude was observed from 35 ms to 50 ms and progressive decline in this increase thereafter. In terms of latency and duration, no significant changes in the first response were observed, while in the second response, the low duration and latency at low ISIs tend to increase as the ISI increases, supporting that the thalamus at low ISIs and the cortex at high ISIs (>140 ms) are effective in shaping the second wave response of the cortex.

Paired-pulse depression has been linked to GABA-B receptor-mediated inhibition in studies [19]. Areas 3b and 1 reflect the postsynaptic neural network activity in the somatosensory cortex, with maximum frequencies around 600 Hz (300–900 Hz). The dominant somatosensory HFO frequency in rats (300–500 Hz) is lower than that of humans (600 Hz) [20]. The early HFOs are thought to be generated by the thalamo-cortical afferents and late HFOs generated by the inhibitor interneurons at area 3b in the parietal region. Similar to N20, the early part of HFO occurs prior to the peak latency of the cortical response, probably consists of the action potential of the thalamo-cortical fibers and is generated as a result of reflection to area 3b (or area 1). On the other hand, the late HFO response starts at around N20, which is the first cortical peak latency and continues up to the last half of the P30, which is the second cortical response [21]. The ratios between the areas tested in this study were also evaluated using a similar approach. While the early portion of HFO reflects the action potential in the thalamo-cortical axon terminal, the late portion probably reflects the burst of the monosynaptic interneurons receiving thalamo-cortical projections and their induction by the inhibition of pyramidal neurons via feedforward. The evidence that inhibitory interneurons in the primary somatosensory cortex have received intense monosynaptic thalamic input in animal studies also supports this suggestion [22]. Similarly, the formation of the second sensory response observed in our study seems to be dependent upon ISI. The early alterations of HFO indicate the impact of the thalamus on the cortex in the second response in low ISIs. In our study, while no significant difference was found in the latency, duration, and mean amplitude of the first response at varying ISIs, a more marked effect of the second response on duration, latency, and amplitude at low ISIs as well as reduction in the rate of increase with increasing ISIs suggest an active role of the thalamus in these processes.

With regard to areas, no significant changes in any of the ISIs were observed in the first response, while detection of significant results particularly at 35 ms in the thalamo-cortical component of the second response is supportive of the notion that the thalamus may have an impact at lower ISIs. The cortical component of the second response reached the cortical component of the first response level at ISI of 50 ms, while the thalamic component of the second response reached thalamic component of the first response level at ISI of 80 ms in our study. The difference in S2/S1 ratio observed between 35, 50 ms and 80, 140 and 500 ms may be due to the thalamic component of the second response. This also supports the notion that the thalamus may have an important role at lower ISIs. On the other hand, significant results for the cortical component of the second response observed for all ISIs are supportive of an effect of the cortex on the thalamus occurring via cortico-thalamic pathways. Analysis of the second/first response area ratios according to HFO showed statistical significance at all ISIs between 150 and 400 Hz, except for 500 ms. These statistical significances suggest that the effect of the first response on the second response is valid up to 500 ms, after which this effect is eliminated.

One limitation of this study is the use of anesthesia in the experimental animals. However, since the recordings were performed with glass electrodes, lack of anesthesia would have been associated with significant challenges. Also, concurrent thalamic recordings could have been performed together with cortical recordings. On the other hand, simultaneous cortical recordings at different ISIs allowed us to observe physiological changings. Our findings may be a basis for future studies of neuropsychiatric disorder models where somatosensory perception is impaired, diseases involving pain models and utilizing different medical treatments.

The present study shows that processing of 2nd sensory responses is dependent on ISIs. Early HFO changes without accompanying late HFO alterations indicate thalamo-cortical drive to cortex plays a major role in determining the 2nd response in low ISIs.

## Figures and Tables

**Table:** The summary of statistical responses the ratio of S2/S1 area for various ISIs and different frequency bands. p denotes the probability values of the t-test, the ration of mean shows the ratio between the average of area under S2 and the average of area under S1.

	Frequency bands				
ISI (msn)	7–13 Hz mean of average	14–50 Hz mean of average	51–150 Hz mean of average	150–400 Hz mean of average	400–800 Hz mean of average
35	0.894	0.351**	0.279**	0.330**	0.757*
50	1.410	0.774**	0.437**	0.433**	1.087
80	0.879	0.911	0.596**	0.632**	1.189
140	0. 21	0.742**	0.515**	0.560**	0.802**
500	0.833	0. 36	0.853	0.878	1.204

* p<0.05,

** p<0.01,

ISI: interstimulus interval.
